# Association Between Dietary Fiber Intake and Risk of Depression in Patients With or Without Type 2 Diabetes

**DOI:** 10.3389/fnins.2022.920845

**Published:** 2022-07-12

**Authors:** Yafei Mao, Xinyuan Li, Shumin Zhu, Yulan Geng

**Affiliations:** Department of Laboratory Medicine, The First Hospital of Hebei Medical University, Shijiazhuang, China

**Keywords:** dietary fiber, depression, National Health and Nutrition Examination, type 2 diabetes (T2D), dietary intake

## Abstract

**Background:**

Depression and type 2 diabetes (T2D) are both serious public health problems, with morbidity and mortality in people increasing year by year, resulting in a heavy economic burden. A correlation between dietary fiber and both has been reported. Nevertheless, few data are available concerning dietary fiber and the risk of depression with or without T2D, which deserve further attention.

**Materials and Methods:**

We assessed the relationship between dietary fiber intake and risk of depression with or without T2D in the 2007–2014 National Health and Nutrition Examination Survey (NHANES) population. A 24-h dietary review was used to assess fiber intake. The Patient Health Questionnaire-9 was used to assess depression. Stability of the results was assessed using restricted cubic spline models and logistic regression, as well as sensitivity analyses.

**Results:**

A total of 17,866 adults aged 20 years and older with a mean age of 49.3 ± 17.7 years were included in this study, of whom 49.5% were male. After adjusting for covariates, the association of dietary fiber intake with the risk of depression appeared to differ between non-T2D group and T2D group (OR, 0.987; 95% CI, 0.979–0.995 vs. OR, 1.003; 95% CI, 0.988–1.017). Furthermore, when dietary fiber was converted to a categorical variable, there was evidence of interaction between T2D status and fiber intake on decreasing the prevalence of depression (*P*-value for interaction = 0.015). Sensitivity analysis showed stable results.

**Conclusion:**

Our findings indicated that whether a patient has T2D may affect the relationship between dietary fiber intake and the risk of depression, which still needs to be confirmed by further randomized controlled trials.

## Introduction

Depression is a frequent mental health condition characterized by a persistently poor mood, a lack of energy, and a loss of interest or pleasure in activities (Battle, 2013). Based on disability-adjusted life years, it has an influence on quality of life and is a major contribution to disease burden (World Health Organization, 2012). The COVID-19 pandemic’s lockdowns, social isolation, economic stress, and other effects have exacerbated the burden of depression (O’Leary, 2021), and serious depressive disorder increases the risk of other diseases and suicide. Given this oncoming fact, it is necessary to explore effective prevention methods for depression. Type 2 diabetes (T2D) has become a serious public health concern, resulting in an increase in accompanying morbidity and mortality, as well as a substantial financial burden (Ding et al., 2014). Microvascular dysfunction is a common phenomenon in diabetic individuals, which might have negative effects for the brain. An increasing number of observational evidence suggests that diabetes-related microvascular dysfunction is linked to an increased risk of cognitive impairment, stroke, and depression (van Sloten et al., 2020). The likelihood of diabetes complications increases with depression (Novak et al., 2016; Vancampfort et al., 2016). Worldwide, approximately half of all diabetic patients suffer from severe depression that has been misidentified by healthcare providers (Saman et al., 2018).

Recently, there is growing evidence that inadequate dietary fiber intake can affect mental health (Smith and Wilds, 2009; Taylor and Holscher, 2020; Hepsomali and Groeger, 2021). Dietary fiber is a kind of plant cell wall that is resistant to human enzyme digestion but may be digested by gut microbial enzymes into short-chain fatty acids (SCFA) (Smith and Wilds, 2009; Taylor and Holscher, 2020). Higher dietary fiber intake has been shown to be a protective factor for depressive symptoms in different study populations (Kim et al., 2020; Khayyatzadeh et al., 2021; Liu et al., 2021; Chrzastek et al., 2022). Also, dietary fiber intake may reduce the risk of newly diagnosed diabetes (Stevens et al., 2002; Yao et al., 2014; Jin et al., 2021). Taylor and Holscher (2020) came to the conclusion that consuming probiotics (a form of dietary fiber) might help with psychological and biological indicators of depression and anxiety. Participants who consumed cereal bars (which contain 1.1 g of dietary fiber each) felt less anxiety than those who did not consume cereal bars, according to Smith and Wilds (2009). Depression is related to dietary factors such as fish (Li et al., 2016), fruits and vegetables (Liu et al., 2016), and some nutrients [folate (Bender et al., 2017), zinc and iron (Li et al., 2017)], according to epidemiological studies. Dietary fiber has been shown to reduce inflammation and the severity of depression symptoms in a recent review article (Swann et al., 2020). The effect of dietary fiber on depression is a complicated topic. It’s difficult to draw definitive conclusions because of potential confounding factors such as the intersection of mechanisms and total diet quality, overweight and obesity, lifestyle factors, and sex (Swann et al., 2020). These studies, however, did not focus on depressed patients with concomitant diabetes. Therefore, the purpose of this study was to investigate the relationship between dietary fiber intake and risk of depression and to explore their relationship between T2D and non-T2D stratification.

Herein, we aimed to compare the association between fiber intake and the risk of depression with or without T2D in a large sample of the general population. To increase the power of the analysis, we used data from four independent cycles of the NHANES from 2007 to 2014.

## Materials and Methods

### Data Sources and Study Population

The National Health and Nutrition Examination Survey (NHANES) is a series of health-related research aimed at determining non-institutionalized Americans’ health and nutritional status (National Health and Nutrition Examination Survey Homepage, 2017). As a representative sample, a multistage, stratified probability strategy was used to select survey participants (Zipf et al., 2013). To capture demographic and health history information, a comprehensive household interview was conducted. A mobile examination center (MEC) was used to conduct physical examinations and collect blood samples. The serum samples were examined at the National Center for Environmental Health’s Division of Laboratory Sciences of the Centers for Disease Control and Prevention.

The National Center for Health Statistics Research Ethics Review Board gave their approval to the project. In our research, NHANES data from 2007 to 2014 were used, and all details were taken from the official website^1^, which is based on public NHANES data. Our study’s participants were above the age of 20 and had completed an interview and evaluation at a MEC. If a participant’s depression status, covariates, or T2D status were missing, they were removed from the study.

### Depressive Symptoms

In the NHANES database, participants were administered the Patient Health Questionnaire (PHQ-9) at inclusion, which was administered as part of the MEC interview questionnaire to assess depression status. It is a nine-item screening tool that assesses the frequency of various depressive symptoms during the previous 2 weeks (Manea et al., 2015). Depression severity can be defined by several cut points from the total score that ranges from 0 to 27, with 0 for “not at all,” 1 for “a few days,” 2 for “more than half of the time,” and 3 for “almost every day.” A score of 10 was used as the cut-off point for inclusion in the depression group, indicating moderate to severe depressive symptoms, and it was shown to be 88% sensitive and specific for major depression (Kroenke et al., 2001).

### Dietary Fiber Intake

Data on dietary fiber intake in the 24 h before the interview were collected through a dietary recall interview conducted at MEC. The Food and Nutrient Database for Dietary Studies (FNDDS) of the United States Department of Agriculture (USDA) was used to calculate nutrient intakes (US Department of Agriculture, 2013). Food code was used to determine the source of dietary fiber. In this study, daily fiber intake was classified into three categorical variables. The 24-h recall approach is most commonly employed to measure nutritional consumption in large-scale surveys like NHANES. Expert consensus at workshops held on a regular basis to evaluate NHANES data collecting procedures led to the decision to employ this method consistently in NHANES over the years (Ahluwalia et al., 2016).

### Identification of Type 2 Diabetes

The definition of T2D cases in the population subgroup was based on two models, namely the American Diabetes Association criteria (American Diabetes Association, 2010) and the self-report questionnaires, which were asked before the physical examination, in the home, using the Computer Assisted Personal Interviewing-CAPI (interviewer administered) system. T2D cases were defined as participants who fulfilled the inclusion criteria:(1) fasting blood glucose (FPG) ≥ 126 mg/dL, (2) 2-h plasma glucose ≥ 200 mg/dL on an oral glucose tolerance test (OGTT), (3) glycated hemoglobin (HbA1c) ≥ 6.5%, and (4) Current use of insulin or diabetes pills to lower blood glucose levels, or a self-report questionnaire that indicates a previous diagnosis of T2D by a physician (Huang et al., 2021).

### Other Covariates

The covariates considered in this study were age, gender, race/ethnicity, body mass index (BMI), education level, poverty to income ratio (PIR), dietary intake, smoking status, drinking status, physical activity, sleep duration, and hypertensive status. A dietary recall interview was conducted in the MEC to collect information on dietary intake in the 24 h prior to the interview, including total dietary food energy, protein, carbohydrate, dietary fiber, vitamin D, and dietary magnesium. According to the standardized calculation method, BMI is weight (kg) divided by height (m) and divided into three categories with cut-off values of 25 and 30 kg/m^2^. Current smokers, former smokers, and never smokers were all separated into three categories (Huang et al., 2021). Participants who had previously smoked ≥ 100 cigarettes and were currently smoking at the time of the interview were classified as “current smokers.” Those who had previously smoked ≥ 100 cigarettes but were no longer smoking were considered as “former smokers.” Participants who had never smoked a cigarette in their lives were labeled as “never smokers.” If a respondent had had at least 12 drinks per year over their lifetime, they were deemed a drinker (Sun et al., 2019). Walking, moderate, and vigorous activities were combined in the physical activity, which included both work and recreational activities. Family income was estimated using the PIR by household size. Hypertension was defined as having a physician’s diagnosis or self-reported current use of anti-hypertensive medication.

### Statistical Analysis

All of the analyses were carried out using the statistical software programs R (^2^ The R Foundation) and Free Statistics software version 1.4 (Yang et al., 2021). Mean ± standard deviation, and frequencies (percentages) were used to describe demographic and clinical data. The t-test was used to analyze the normal distribution and the Kruskal-Wallis test to analyze the skewed distribution in continuous variables. Univariate and multivariate logistic regression were used to investigate the relationship between dietary fiber intake and risk of depression. In multivariate logistic regression, dietary fiber was analyzed as a continuous and categorical variable, with model 1 adjusting for age and gender and model 2 adjusting for variables from model 1 as well as clinical indications including BMI, race/ethnicity, education, smoking status, alcohol consumption, PIR, diabetes, hypertension, and total daily energy intake. More importantly, the relationship between participants’ dietary fiber intake levels and risk of depression was compared between the non-T2D and T2D group. Interaction among subgroups was inspected by the likelihood ratio test. Sensitivity analyses were performed by excluding outliers with dietary fiber intake outside the mean ± 2 SD (0–37.502 g/d) or mean ± 3 SD (0–47.963 g/d) ranges. *P*-value < 0.05 were considered statistically significant.

## Results

### Baseline Characteristics of the Study Population

**FIGURE 1:**
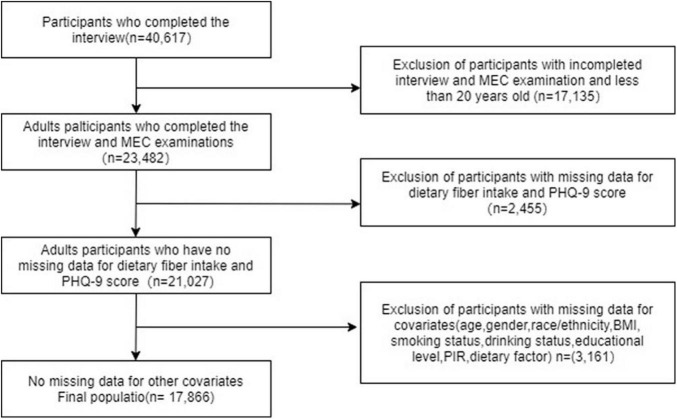
Flowchart of participant selection.

This study selected 40,617 potential participants from NHANES (2007–2014), of which 23,482 adults (≥20 years) completed interviews and underwent MEC screening for inclusion in our study. Participants with missing PHQ-9 scores and dietary fiber intake (*n* = 2,455) were excluded. The remaining 17,866 participants were included in our analysis after excluding those with missing covariate data (*n* = 3,161). Figure 1 depicts a flow chart of the exclusion criteria. Table 1 shows the baseline characteristics of the research participants based on their T2D status as defined by the American Diabetes Association criteria (American Diabetes Association, 2010). In this sample, males, less educated, smokers, hypertensives, and those with lower levels of work activity and recreational activity were more likely to have combined T2D. Participants with T2D had significantly lower daily dietary intakes of energy, protein, carbohydrate, fiber, and magnesium and lower PIR than those without T2D. Besides, participants in the group with T2D were older and possessed a higher BMI.

**TABLE 1:** Baseline characteristics of participants.

Covariates	Total	Without T2D	With T2D	*P*-value
	(*n* = 17,866)	(*n* = 14,666)	(*n* = 3,200)	
Age (years)	49.3 ± 17.7	46.7 ± 17.5	61.0 ± 13.5	<0.001
Gender, *n* (%)				0.007
Female	9,019 (50.5)	7,473 (51)	1,546 (48.3)	
Male	8,847 (49.5)	7,193 (49)	1,654 (51.7)	
Education level, *n* (%)				<0.001
Did not graduate from high school	4,327 (24.2)	3,207 (21.9)	1,120 (35)	
Graduated from high school	4,092 (22.9)	3,341 (22.8)	751 (23.5)	
College education or above	9,447 (52.9)	8,118 (55.4)	1,329 (41.5)	
Race/Ethnicity, *n* (%)				<0.001
Mexican American	2,531 (14.2)	2,011 (13.7)	520 (16.2)	
Other Hispanic	1,681 (9.4)	1,350 (9.2)	331 (10.3)	
Non-Hispanic white	8,372 (46.9)	7,073 (48.2)	1,299 (40.6)	
Non-Hispanic black	3,704 (20.7)	2,887 (19.7)	817 (25.5)	
Other races	1,578 (8.8)	1,345 (9.2)	233 (7.3)	
PIR, Mean ± SD	2.5 ± 1.6	2.6 ± 1.7	2.3 ± 1.5	<0.001
**Dietary factors**				
Energy (kcal)	2112.0 ± 1007.4	2164.5 ± 1022.8	1871.1 ± 895.5	<0.001
Protein (gm)	81.2 ± 43.0	82.4 ± 43.5	75.8 ± 39.7	<0.001
carbohydrate (gm)	256.5 ± 128.1	263.8 ± 130.4	222.9 ± 111.2	<0.001
Fiber (gm)	16.6 ± 10.4	16.7 ± 10.5	15.9 ± 10.0	<0.001
Vitamin D (mg)	3.1 (1.2, 6.0)	3.2 (1.2, 6.1)	3.1 (1.3, 5.6)	0.107
Magnesium (mg)	294.5 ± 149.9	299.4 ± 152.4	272.3 ± 135.5	<0.001
BMI (kg/m^2^), Mean ± SD	29.2 ± 6.9	28.4 ± 6.6	32.5 ± 7.5	<0.001
BMI, *n* (%)				<0.001
<25 kg/m^2^	5,195 (29.1)	4,769 (32.5)	426 (13.3)	
25∼30 kg/m^2^	5,923 (33.2)	5,007 (34.1)	916 (28.6)	
≥30 kg/m^2^	6,748 (37.8)	4,890 (33.3)	1858 (58.1)	
Glycohemoglobin (%)	5.7 ± 1.1	5.4 ± 0.4	7.2 ± 1.7	<0.001
Fasting glucose (mg/dL)	108.3 ± 34.6	97.8 ± 9.7	148.3 ± 58.1	<0.001
Two hour glucose (OGTT) (mg/dL)	120.6 ± 51.8	109.2 ± 31.9	223.5 ± 77.0	<0.001
Smoking status, *n* (%)				<0.001
Current smoker	4,398 (24.6)	3,301 (22.5)	1,097 (34.3)	
Former smoker	3,796 (21.2)	3,260 (22.2)	536 (16.8)	
Never smoker	9,672 (54.1)	8,105 (55.3)	1,567 (49)	
Drinking status, *n* (%)				<0.001
≥ 12 alcohol drinks a year (%)	13,061 (73.1)	10,989 (74.9)	2,072 (64.8)	
Physical activity, *n* (%)				
Vigorous work activity	3,343 (18.7)	2,933 (20)	410 (12.8)	<0.001
Moderate work activity	6,466 (36.2)	5,541 (37.8)	925 (28.9)	<0.001
Walk or bicycle	4,658 (26.1)	4,031 (27.5)	627 (19.6)	<0.001
Vigorous recreational activities	3,779 (21.2)	3,546 (24.2)	233 (7.3)	<0.001
Moderate recreational activities	7,281 (40.8)	6,290 (42.9)	991 (31)	<0.001
Hypertension, *n* (%)	6,485 (36.3)	4,381 (29.9)	2104 (65.8)	<0.001
Depression, *n* (%)	1,670 (9.3)	1,238 (8.4)	432 (13.5)	<0.001
Sleeping time, *n* (hours)	6.8 ± 1.4	6.8 ± 1.4	6.8 ± 1.6	0.088

*Data presented are ORs and 95% CIs.*

*T2D, type 2 diabetes; PIR, ratio of family income to poverty; BMI, body mass index.*

### Dietary Fiber Intake Affects the Risk of Depression Patients Without Type 2 Diabetes

Univariate analysis showed that age, gender, education level, PIR, BMI, physical activity, smoking status, diabetes, hypertension, and dietary intake such as total energy, fiber, protein, vitamin D, and magnesium were associated with the risk of depression (Table 2). The findings of the multifactorial logistic regression analysis are shown in Table 3. In the unadjusted model, dietary fiber intake was shown to be inversely related to the risk of depression (OR, 0.97; 95% CI, 0.97–0.98). Results were similar after adjusting for age and gender (OR, 0.98; 95% CI, 0.97–0.98). After adjusting for other possible confounders, including race/ethnicity, BMI, smoking status, alcohol consumption, education level, PIR, diabetes, hypertension, and total daily energy intake, the negative association remained significant (all *P*-value < 0.001). This significant relationship was also present in the different adjusted models when dietary fiber was transformed into a categorical variable (all *P*-value < 0.005). After adjusting for age, gender, BMI, race/ethnicity, educational level, PIR, smoking status, alcohol consumption, physical activity, hypertension and total daily energy intake, increasing total dietary fiber intake significantly reduced the incidence of depression in the group without T2D (OR, 0.987; 95% CI, 0.979–0.995), but not in the T2D group (OR, 1.003; 95% CI, 0.988–1.017). There was an interaction between dietary fiber and depression risk in patients with T2D and those without T2D when dietary fiber intake was transformed into a categorical variable (*P*-value for the likelihood ratio test for the interaction was *P* = 0.015) (Table 4).

**TABLE 2:** Association of covariates and depression risk.

Variable	OR_95 CI%	*P*-value
Age (years)	0.996 (0.994∼0.999)	0.013
**Gender, *n* (%)**		
Female	1 (reference)	
Male	0.522 (0.470∼0.580)	<0.001
**Education level, *n* (%)**		
Did not graduate from high school	1 (reference)	
Graduated from high school	0.663 (0.580∼0.759)	<0.001
College education or above	0.465 (0.414∼0.522)	<0.001
**Race/Ethnicity, *n* (%)**		
Mexican American	1 (reference)	
Other Hispanic	1.408 (1.159∼1.711)	<0.001
Non-Hispanic white	0.947 (0.814∼1.103)	0.486
Non-Hispanic black	0.998 (0.840∼1.185)	0.980
Other races	0.650 (0.510∼0.827)	<0.001
PIR, *n* (%)	0.657 (0.632∼0.683)	<0.001
**Dietary factors**		
Energy (kcal)	1 (1∼1)	<0.001
Protein (gm)	0.993 (0.992∼0.994)	<0.001
Carbohydrate (gm)	1.000 (0.999∼1.000)	0.187
Fiber (gm)	0.971 (0.965∼0.977)	<0.001
Vitamin D (mg)	0.973 (0.962∼0.985)	<0.001
Magnesium (mg)	0.998 (0.998∼0.999)	<0.001
BMI, *n* (%)	1.038 (1.031∼1.045)	<0.001
**Smoking status, *n* (%)**		
Current smoker	1 (reference)	
Former smoker	2.250 (1.962∼2.58)	<0.001
Never smoke	0.825 (0.722∼0.942)	0.005
**Drinking status, *n* (%)**		
≥12 alcohol drinks a year (%)	1.011 (0.902∼1.132)	0.856
**Physical activity, *n* (%)**		
Vigorous work activity	0.938 (0.823∼1.07)	0.340
Moderate work activity	0.800 (0.718∼0.891)	<0.001
Walk or bicycle	0.852 (0.756∼0.959)	0.008
Vigorous recreational activities	0.395 (0.336∼0.465)	<0.001
Moderate recreational activities	0.457 (0.408∼0.513)	<0.001
Sleeping time, *n* (%)	0.752 (0.726∼0.779)	<0.001
Diabetes, *n* (%)	1.693 (1.506∼1.903)	<0.001
Hypertension, *n* (%)	1.673 (1.512∼1.851)	<0.001

*Data presented are ORs and 95% CIs.*

*PIR, the ratio of family income to poverty; BMI, body mass index.*

**TABLE 3:** Weighted odds ratios (95% confidence intervals) of depression and different dietary fiber intake levels in different models.

Dietary fiber intake (mg/d)	Cases/participants	Non-adjusted model	Model 1	Model 2
Total fiber	1670/17866	0.97 (0.97∼0.98)	0.98 (0.97∼0.98)	0.99 (0.98∼0.99)
*P*-value		<0.001	<0.001	<0.001
**Subgroups**				
Quartile 1	736/5,879	1.00 (Ref.)	1.00 (Ref.)	1.00 (Ref.)
Quartile 2	520/6,023	0.66 (0.59∼0.74)	0.68 (0.60∼0.76)	0.84 (0.74∼0.95)
Quartile 3	414/5,964	0.52 (0.46∼0.59)	0.58 (0.51∼0.66)	0.79 (0.67∼0.92)
*P*-trend		<0.001	<0.001	0.002

*Data presented are ORs and 95% CIs.*

*Model 1: adjusted for age and gender.*

*Model 2: additionally adjusted for BMI, race/ethnicity, educational level, PIR, smoking status, alcohol consumption, diabetes, hypertension, and total daily energy intake.*

**TABLE 4:** Interactive effect of dietary fiber intake and depression in patients with or without type 2 diabetes (T2D).

Variable	Without T2D (*n* = 14,666)		With T2D (*n* = 3,200)		*P* for interaction
	OR 95% CI	*P*-value	OR 95% CI	*P*-value	
Dietary fiber intake (g/d)	0.987 (0.979∼0.995)	0.002	1.003 (0.988∼1.017)	0.739	0.188
Subgroups					
Quartile 1	1.000 (Ref)		1.000 (Ref)		0.015
Quartile 2	0.766 (0.659∼0.890)	<0.001	1.276 (0.977∼1.665)	0.073	
Quartile 3	0.755 (0.632∼0.903)	0.002	1.238 (0.888∼1.725)	0.207	
Trend test		<0.001		0.159	

*Data presented are ORs and 95% CIs.*

*Adjusted for age, gender, BMI, race/ethnicity, educational level, PIR, smoking status, alcohol consumption, physical activity, hypertension, and total daily energy intake.*

### Threshold Effect Analysis and Sensitive Analysis

**FIGURE 2:**
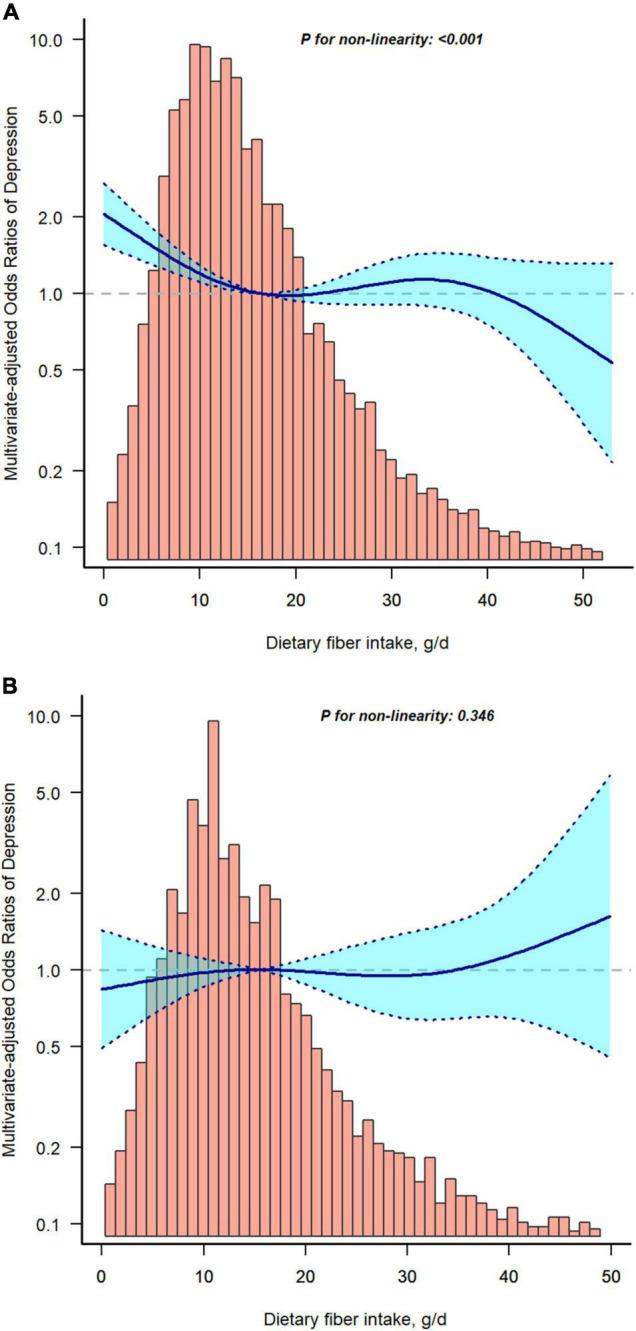
Restricted cubic spline model of the odds ratios of dietary fiber intakes with depression without type 2 diabetes (T2D) **(A)** or with T2D **(B)**. Adjusted for age, gender, race/ethnicity, body mass index (BMI), educational level, poverty to income ratio (PIR), smoking status, alcohol consumption, physical activity, hypertension, and total daily energy intake. The dashed lines represent the 95% confidence intervals.

Restricted cubic spline analysis was applied to investigated the dose-response association between fiber intake and risk of depression with or without T2D. Figure 2 shows a non-linear relationship between fiber intake and depression without T2D (A), but not in T2D group (B). To support our conclusions, we conducted sensitivity analyses. The results remained stable after excluding participants with dietary fiber intakes > 47.963 g/d (mean ± 3SD) (Supplementary Table S1). The risk of depression decreased significantly with increasing dietary fiber intake in the non-diabetic group (OR, 0.986; 95% CI, 0.978–0.995, *P* = 0.003), whereas the relationship was not significant in the T2D group (OR, 1.002; 95% CI, 0.986–1.018, *P* = 0.805). When dietary fiber was transformed into a categorical variable, the interaction of dietary fiber intake on the prevalence of depression with or without T2D was significant (*P*-value for the likelihood ratio test for the interaction was *P* = 0.017). When subjects with dietary fiber intakes > 37.502 g/d (mean ± 2SD) were excluded (Supplementary Table S2), the incidence of depression was significantly lower in the no-diabetes group as dietary fiber increased (OR, 0.985; 95% CI, 0.975–0.994, *P* = 0.002). However, the decrease in the T2D group was not significant (OR, 1.002; 95% CI, 0.984–1.020, *P* = 0.841). Similarly, the interaction likelihood ratio test had a *P*-value of 0.019 when dietary fiber was turned into a categorical variable.

## Discussion

From this cross-sectional analysis of United States adults (≥20 years) using NHANES (2007–2014) data, we found a negative association between dietary fiber intake and risk of depression when adjusted for potentially important confounders, and a non-linear negative relationship in the non-T2D group. As far as we understand, this is the first research to explore the relationship between fiber intake and risk of depression in patients with or without T2D in a population of US adults.

In our study, we found a negative association between dietary fiber intake and depressive symptoms, which is consistent with the results of previous studies (Woo et al., 2006; Gangwisch et al., 2015; Kim et al., 2015, 2020; Khayyatzadeh et al., 2021; Liu et al., 2021; Chrzastek et al., 2022). The Women’s Health Initiative Observational Study (Gangwisch et al., 2015) showed that among postmenopausal women, higher dietary fiber consumption was associated with a decrease in OR for depressive symptoms. A Korean study (Kim et al., 2015) of adolescent girls showed that dietary fiber intake reduced the risk of depressive symptoms. Similar results were found in an elderly Chinese population (Woo et al., 2006). According to a cross-sectional study (Liu et al., 2021), increased dietary fiber intake was a protective factor toward depression and anxiety in hypertension individuals. In contrast, Gopinath et al. (2016) found no statistically significant association between depression and dietary fiber intake in vegetables, fruit and bread. In addition, a large number of studies (Yao et al., 2014; Jin et al., 2021; Kimura et al., 2021) have shown a negative association between dietary fiber intake and diabetes risk, but few have focused on the relationship between dietary fiber intake and depression with or without T2D.

Although little is known about the mechanisms by which dietary fiber affects depression, several possibilities have been reported. First, dietary fiber can change the composition of the gut microbiota (Albenberg and Wu, 2014), which can influence brain function by connecting with the central nervous system and regulating inflammation, oxidative stress (Selhub et al., 2014), the serotonergic system (O’Mahony et al., 2015), and the stress response (Moloney et al., 2014). Second, short-chain fatty acids generated by dietary fiber fermentation are thought to modulate the inflammatory response (Maslowski et al., 2009), which is an important mediator in depression (Berk et al., 2013). Third, postprandial hyperglycemia may cause an increase in oxidative stress, whereas dietary fiber reduces postprandial plasma glucose, which might help to decrease inflammation (Chandalia et al., 2000; Qi and Hu, 2007). By controlling intestinal flora, dietary fiber has been shown to improve unpleasant feelings (Koh et al., 2016), consequently improving the intestinal–brain axis in depressed patients. Thus, Xu et al. (2018) investigated 40,617 individuals in the United States and concluded that total fiber, vegetable fiber, and fruit fiber intakes were all negatively related to depressed symptoms. Dietary fiber intake was revealed to be a protective factor for depression without T2D (*P* < 0.05) in our study, and this finding may not be applicable to depression patients with T2D.

In this research, the association between dietary fiber and depression in T2D and non-T2D patients was explored after adjusting for potential variables such as baseline characteristics and total energy intake. This research provides several strengths. First, we employed a large, nationally representative sample of adults in US. Second, we took into account and adjusted for known and potential depressed symptom risk variables. Third, the researchers looked at relationships stratified by T2D and conducted sensitivity analyses on dietary fiber intake. In addition, we also performed a dose-response analysis to assess the relationship between fiber intake and depression in different T2D status.

However, there are some clear limitations to this study that should be considered. Firstly, causal inferences cannot be drawn from a cross-sectional study. Secondly, because the interview data for this study was self-reported, there is a risk of misunderstanding of the questions or recall problems. Biases in recall and self-report may exist since the dietary data were derived from a self-reported 24-h dietary review. The results may be inaccurate if the same participant is resampled in a different circle. However, the chances of it happening are exceedingly low because participants were recruited using a multi-stage, stratified probability design and NHANES analyzes around 5,000 people each year in 15 different counties throughout the country, a large population base in the United States. Thirdly, despite adjusting for various confounding factors, we cannot rule out the potential that the observed relationships are attributable to unmeasured confounders. Finally, our study population seemed to be younger in the non-T2D population, and there are some differences in baseline characteristics between two groups; however, we have adjusted for most of the relevant variables in logistic regression models, including some dietary factors, etc., and we used a large sample, especially in the non-T2D population. Given these limitations, we need well-designed multicenter randomized controlled trials to validate our findings.

## Conclusion

Overall, we found a non-linear negative relationship between dietary fiber intake and the risk of depression in the non-T2D group. Furthermore, whether or not a person has T2D may have an impact on the link between dietary fiber consumption and depression risk. More prospective studies are necessary to investigate the causal association between fiber intake and depression risk in patients with and without T2D.

## Data Availability Statement

The raw data supporting the conclusions of this article will be made available by the authors, without undue reservation.

## Ethics Statement

The studies involving human participants were reviewed and approved by CDC’s National Center for Health Statistics Institutional Research Ethics Review Board. The patients/participants provided their written informed consent to participate in this study.

## Author Contributions

YM designed, analyzed, and wrote the manuscript. XL was responsible for data collection. SZ performed the data interpretation. YG reviewed and revised the manuscript. All authors contributed to the manuscript and approved the submitted version.

## Conflict of Interest

The authors declare that the research was conducted in the absence of any commercial or financial relationships that could be construed as a potential conflict of interest.

## Publisher’s Note

All claims expressed in this article are solely those of the authors and do not necessarily represent those of their affiliated organizations, or those of the publisher, the editors and the reviewers. Any product that may be evaluated in this article, or claim that may be made by its manufacturer, is not guaranteed or endorsed by the publisher.

## Footnotes

1
https://www.cdc.gov/nchs/nhanes/aboutnhanes.htm

2
http://www.R-project.org
