# Supplementary material for: Association Between Dietary Fiber Intake and Risk of Depression in Patients With or Without Type 2 Diabetes
Source: Front Neurosci. 2022 Jul 12;16:920845. doi: 10.3389/fnins.2022.920845 (PMC9642095; doi:10.3389/fnins.2022.920845)
Supplement: Supplementary file 1 [file Table_1.DOCX]

**Supplementary Table S1.** Interactive effect of dietary fiber intake and depression in patients with or without T2D. Dietary Fiber exclude（X±3SD）

| **Variable** | **Without T2D (n=14440)** | |  | **With T2D (n=3161)** | | P for interaction |
| --- | --- | --- | --- | --- | --- | --- |
|  | **OR 95% CI** | ***P*-value** |  | **OR 95% CI** | ***P*-value** |  |
| Dietary fiber intake (g/d) | 0.986 (0.978~0.995) | 0.003 |  | 1.002(0.986~1.018) | 0.805 | 0.323 |
| Subgroups |  |  |  |  |  |  |
| Quartile 1 | 1.000(Ref) |  |  | 1.000(Ref) |  | 0.017 |
| Quartile 2 | 0.755 (0.648~0.878) | <0.001 |  | 1.293 (0.990~1.690) | 0.060 |  |
| Quartile 3 | 0.746 (0.623~0.893) | 0.001 |  | 1.250 (0.895~1.747) | 0.191 |  |
| Trend test |  | <0.001 |  |  | 0.142 |  |

*Notes: Data presented are ORs and 95% CIs.*

*Adjusted for age, gender, BMI, race/ethnicity, educational level, PIR, smoking status, alcohol consumption, physical activity, hypertension and total daily energy intake.*
