# Supplementary material for: Association Between Dietary Fiber Intake and Risk of Depression in Patients With or Without Type 2 Diabetes
Source: Front Neurosci. 2022 Jul 12;16:920845. doi: 10.3389/fnins.2022.920845 (PMC9642095; doi:10.3389/fnins.2022.920845)
Supplement: Supplementary file 2 [file Table_2.DOCX]

**Supplementary Table S2.** Interactive effect of dietary fiber intake and depression in patients with or without T2D. Dietary Fiber exclude（X±2SD）

| **Variable** | **Without T2D (n=14025)** | |  | **With T2D (n=3092)** | | P for interaction |
| --- | --- | --- | --- | --- | --- | --- |
|  | **OR 95% CI** | ***P*-value** |  | **OR 95% CI** | ***P*-value** |  |
| Dietary fiber intake (g/d) | 0.985 (0.975~0.994) | 0.002 |  | 1.002 (0.984~1.020) | 0.841 | 0.433 |
| Subgroups |  |  |  |  |  |  |
| Quartile 1 | 1.000(Ref) |  |  | 1.000(Ref) |  | 0.019 |
| Quartile 2 | 0.756 (0.649~0.880) | <0.001 |  | 1.323 (1.011~1.730) | 0.041 |  |
| Quartile 3 | 0.751 (0.625~0.901) | 0.002 |  | 1.275 (0.908~1.790) | 0.161 |  |
| Trend test |  | <0.001 |  |  | 0.110 |  |

*Notes: Data presented are ORs and 95% CIs.*

*Adjusted for age, gender, BMI, race/ethnicity, educational level, PIR, smoking status, alcohol consumption, physical activity, hypertension and total daily energy intake.*
